# Sulfate Radical in (Photo)electrochemical Advanced Oxidation Processes for Water Treatment: A Versatile Approach

**DOI:** 10.1021/acs.jpclett.3c01361

**Published:** 2023-09-28

**Authors:** Babatunde A. Koiki, Charles Muzenda, Kehinde D. Jayeola, Minghua Zhou, Frank Marken, Omotayo A. Arotiba

**Affiliations:** † Department of Chemical Sciences, University of Johannesburg, Doornfontein 2028, Johannesburg, South Africa; ‡ Key Laboratory of Pollution Process and Environmental Criteria, Ministry of Education, College of Environmental Science and Engineering, Nankai University, Tianjin 300350, China; § Department of Chemistry, University of Bath, Claverton Down, Bath BA2 7AY, U.K.; ∥ Centre for Nanomaterials Science Research, University of Johannesburg,Johannesburg 2028, South Africa

## Abstract

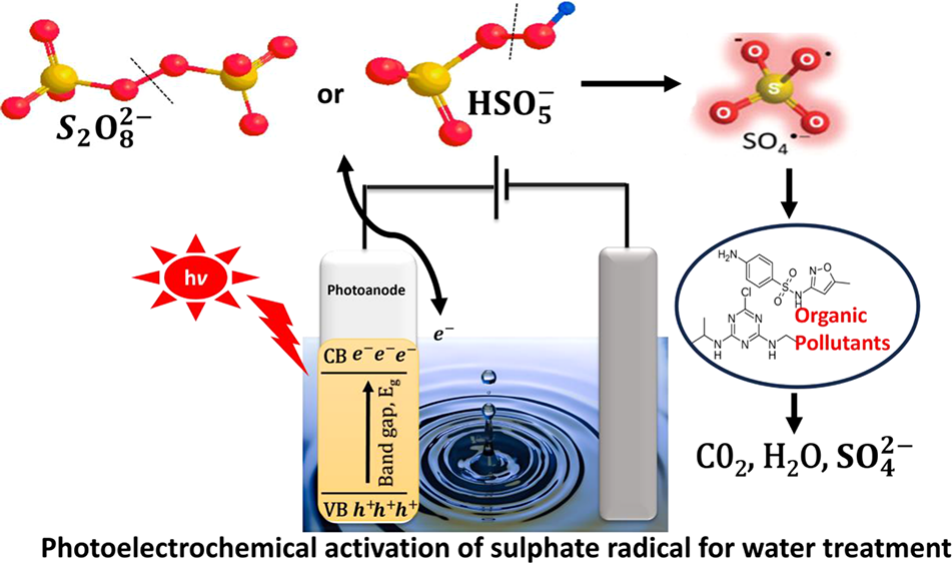

The search for a simple and clean approach toward the production of sulfate radicals for water treatment gave rise to electrochemical and photoelectrochemical activation techniques. The photoelectrochemical activation method does not just distinguish itself as a promising activation method, it is also used as an efficient water treatment method with the ability to treat a myriad of pollutants due to the complementary effects of highly reactive oxidizing species. This perspective highlights some merits that distinguish sulfate monoanion radicals from hydroxyl radicals. It highlights the electrochemical, photoelectrochemical, and in situ photoelectrochemical routes of generating sulfate radicals for advanced oxidation process approach to water treatment. We provide a detailed account of the few known applications of sulfate radical enhanced photoelectrochemical treatments of water laden with organics. Finally, we placed this area of research in perspective by providing outlooks and conclusive remarks.

In recent years, the detrimental effects arising from emerging organic pollutants have continued to receive attention. Some of these emerging pollutants are known to be toxic, recalcitrant, and nonbiodegradable. Therefore, the search for low cost, efficient, versatile, and sustainable technology is of great essence.^1,2^ Advanced oxidation processes (AOPs) have proven to be a group of efficient methods for the treatment of emerging recalcitrant and toxic organics due to the in situ production of hydroxyl radicals which are nonspecific and can completely degrade a myriad of organic pollutants in water.^3−6^ Generally, the known AOPs include chemical, photochemical, and photocatalytic methods like H_2_O_2_/UVC radiation, ozone and ozone-based techniques, photocatalysis, Fenton’s and photo-Fenton-based techniques.^7−11^ The introduction of electrochemical processes into AOP gives rise to another subset of AOP called electrochemical advanced oxidation processes (EAOPs), which are a promising set of electrochemistry driven methods for wastewater treatment. EAOPs possess remarkable advantages: (i) the ability to work under mild temperature and pressure; (ii) ease of operation; (iii) energy efficiency; (iv) environmentally friendliness; and (v) the ability to mineralize a wide range of recalcitrant organic pollutants in wastewater.^12,13^ Photoelectrochemical or photoelectrocatalytic degradation are EAOPs that combine electrochemical and photocatalytic oxidation. This contrasts with photocatalysis in the following ways. (i) Ease of photocatalyst recovery: In photocatalysis, catalyst recovery can be labor-intensive, but since the photocatalyst is immobilized on a substrate in photoelectrocatalysis, recovery is easier. (ii) Ease of recycling: Since photocatalyst recovery is laborious in photocatalysis, recycling will also be a strenuous exercise. However, in photoelectrocatalysis, the photocatalyst is easily recycled. (iii) The introduction of an external bias potential in the photoelectrocatalysis promotes separation of photogenerated charge carriers thereby suppressing rapid recombination as compared to processes in photocatalysis.^14,15^ (iv) The synergistic effects of photocatalysis and electrocatalysis position photoelectrocatalysis as advantageous. (v) In a mild environment, photoelectrocatalysis possesses a high power of oxidation–reduction reactions when the semiconductor is irradiated, thereby making it suitable for the redox conversion of the contaminants in aqueous medium.^16^

Photoelectrocatalytic systems based on the production of hydroxyl radical at the photoanode have been developed with many types of semiconductors (with sufficient band edge energy) and for the treatment of organic pollutants of different classes such as dyes, pharmaceuticals etc. Hydroxyl radical based photoelectrocatalysis systems are designed for a wide range of applied potentials and time. An applied potential of 3 V vs NHE and a reaction time of 2 to 4 h are not uncommon in the literature. The new opportunity in hydroxyl-based photoelectrocatalysis is the use of visible light toward improved sustainability. The semiconductor(s) need to be tuned using approaches such as doping, heterojunction formation, and exploiting synthesis routes to change the morphology of the material.

The use of radical species in oxidizing or degrading recalcitrant pollutants has been proven a powerful approach to water treatment. Relying only on active surface states on semiconductor surfaces is insufficient, and the formation of radical species in solution really enhances treatment performance. While OH radical generation AOPs or EAOPs are the most studied, the authors feel that other radicals such as in particular sulfate radicals can be explored further. After all, our goal is to improve the efficiency of AOP and EAOP systems for water treatment be it in reduced degradation time, reduced energy costs or simpler material.

Sulfate radical-based advanced oxidation processes have the following merits: (i) sulfate radicals diffuse rapidly and possess a longer lifetime of ∼300 μs (environment dependent) as compared to hydroxyl radicals (∼40 μs or less).^17^ This suggests that sulfate radicals will survive longer than hydroxyl radicals in an aqueous solution, thereby potentially degrading more pollutants. (ii) Depending on the pH of the solution, sulfate radicals possess a higher redox potential *E*_o_ = 2.5–3.1 V vs NHE compared to that of hydroxyl radicals (*E*_o_ = 1.8–2.7 V vs NHE).^18^ In particular, in alkaline media, the balance is on the side of OH radicals. This suggests that the higher oxidizing power possessed by the sulfate radicals can promote the abatement of recalcitrant toxic organic pollutants. (iii) When present in neutral to basic medium, sulfate radicals exhibit outstanding performance and notable stability, thereby lending it a wide range of applications.^19^ (iv) Sulfate radical processes can simultaneously produce secondary oxidants such as hydroxyl radicals, superoxides, and singlet oxygen thereby enhancing the rate and extent of degradation. (v) In terms of reaction kinetics, sulfate radicals easily and rapidly react with organic molecules nearly following second order kinetics between 10^5^ and 10^9^ M^–1^ s^–1^ but can also dimerize to peroxodisulfate.^20^ (vi) In a complex water matrix, sulfate radicals have been reported to possess higher selectively and reactivity toward organic pollutant degradation compared with hydroxyl radicals.^21^ (vii) Sulfate radical based processes have been employed in the treatment of groups of contaminants that are “difficult to degrade”.^22−24^

The above merits suggest that sulfate radical based processes have the potential to compete, complement or outperform the more popular hydroxyl radicals-based advanced oxidation process.^25^ Surprisingly, sulfate radical-promoted photoelectrochemical degradation of recalcitrant organics has not been extensively investigated in recent times. Thus, we provide our perspective on the electrochemical, photoelectrochemical, and in situ photoelectrochemical methods of generating sulfate radicals. We aim to furnishing the research community with some information on the fundamentals and application of sulfate radical generation and sulfate radical based processes for water treatment. We hope to stimulate interest in the exploration of sulfate radical based processes, especially, electrochemistry-based approaches for the removal of recalcitrant organic pollutants from wastewater. Recent research in sulfate radical based AOPs is presented and discussed with an outlook and conclusive remark.

Sulfate radicals can be produced by activating salts such as peroxymonosulfate (PMS, HSO_5_^–^) and persulfate or peroxydisulfate (PS or PDS, S_2_O_8_^2–^). PMS consist of potassium hydrogen sulfate, potassium sulfate, and potassium peroxymonosulfate in the ratio 1:1:2. PS/PDS contain both sulfate ions and positive ions.^1,26^ There is the presence of O–O bond similar to that of hydrogen peroxide in PMS and PS. Due to its long bond distance of about 1.497 Å and low bond energy of about 33.5 kcal/mol, the O–O can be cleaved through a series of oxidizing processes to activate PS.^27,28^

Several methods of generating sulfate radicals by the activation of PS and PMS involving the use of heat,^29^ UV,^30^ transition metals,^31^ alkaline,^32^ carbon-based materials^28^ have been reported. These methods suffer some drawbacks as shown in Table 1, hence the need for other methods that can overcome these significant limitations.

**Table 1:** Various Activation Methods and Their Drawbacks

Activation Methods	Drawbacks	Ref
Heat	i. High energy input is required, which therefore can be challenging on industrial scale.	(2, 33)
	ii. High temperature is also required, thus leading to side reactions.	
	iii. There is a possibility of the sulfate radicals produced to be readily transformed into hydroxyl radicals.	
	iv. PEC for water treatment is usually a low energy consuming approach that takes place under mild temperatures and pressure. Since high energy and temperature are required to drive the thermal activation of sulfate radicals, this approach is unsuitable in PEC.	
Alkaline	i. It possesses low efficiency resulting in extended degradation time.	(2, 33, 34)
	ii. It cannot be employed when degrading polycyclic aromatic hydrocarbons.	
	iii. Since a very high pH is needed to drive the system, there is a possibility that the process may affect the speciation of metals.	
	iv. There is a need to combine this method with other activation methods to boost its performance of efficient degradation of organic pollutants.	
UV	i. The persulfate activation for efficient pollutant degradation is highly dependent on a wavelength of 254 nm, hence not suitable for a wider wavelength range.	(35, 36)
	ii. The method is not sustainable as the wavelength required for activation of PS and PMS in recent times has stretched toward visible light and sunlight to avoid the more expensive and hazardous UV light.	
Carbon-based materials	i. Carbon-based materials are prone to surface deactivation and, hence may lose their capacity to activate PS and PMS	(2)
Transition metal ions	i. For a homogeneous system, recovery of metal ions is very difficult. Also, effluents containing elevated amounts of organics will require high amounts of metal ions in the wastewater. In addition, metal ions are likely to precipitate in the basic medium and become hydrated in the acidic medium, thus impeding the performance of the metal ions in activating PS and PMS.	(1, 2)
	ii. For a heterogeneous system, the ability to activate PS and PMS depends majorly on the excellent properties of the materials such as their surface properties, morphologies, etc. This suggests it will be material selective.	

*Electrochemical Activation.* The search for a simple and clean technique to activate persulfate ions to produce sulfate radicals has led to an electrochemical activation approach that is environmentally friendly and controllable.^37,38^ When persulfate salt is activated electrochemically, it may lead to nonradical oxidation with high selectivity toward certain pollutants and reduce the production of byproducts that are hazardous. Interestingly, researchers have shown that the electrochemical activation route of producing sulfate radicals can achieve total mineralization of different persistent organic pollutants that cannot be degraded by activated persulfate alone.^39,40^ According to the literature, the various effective routes to generating sulfate radicals include the following:^41^

i. Electrochemical activation of sulfate ions via a direct electron transfer. This takes place at the surface of the anode and this particular route has been majorly associated with boron-doped diamond,^42,43^ blue titanium dioxide,^44^ and self-doped titanium dioxide nanotube arrays^45^ as shown in eq 1.
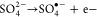
1

ii. Anodic electrogeneration of persulfate ion at large oxygen-over potential anodes like diamond,^42,46^ PbO_2_,^47^ and Ti_4_O_7_.^48^ Here, sulfate ion is indirectly oxidized in the presence of heterogeneous free hydroxyl radicals to produce sulfate radicals as represented in 2.
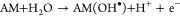
2Where AM = anodic material
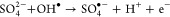
3
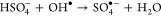
4
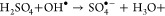
5

Worthy of note is the fact that the formation of sulfate radicals depends on the sulfate used as the starting material.^46,49^

iii. Direct cathodic reduction of persulfate at certain electrocatalytic surfaces as shown in eq 6.^50^
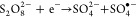
6

For example, platinum, graphite, and Fe_3_O_4_ modified glassy carbon electrodes have been used for the cathodic reduction of persulfate ions to sulfate radicals.^37,51^

iv. The addition of persulfate into the solution as a starting material. Herein, the sulfate radical is produced via heterogeneous activation using a sacrificial iron anode as shown in eq 7.
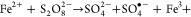
7

Electrochemical activation routes open the possibilities of in situ generation or simultaneous generation of sulfate radical during the photoelectrochemical oxidation process.

*Photoelectrochemical Activation.* The basic principle of the photoelectrochemical process involves the combination of photocatalytic and electrochemical processes in the presence of a bias potential. In the presence of visible or solar light, semiconductor photocatalysts undergo electronic transitions to generate charge carriers that are mostly electrons and holes. The electrons from the valence band of the semiconductor migrate to the conduction band, thereby resulting in the generation of holes in the valence band. These electrons in the conduction band contribute toward photoreduction, while the photogenerated holes in the valence band are responsible for photo-oxidation. In the process of photoreduction, the PS and PMS gain an electron from the conduction band (Figure 1) resulting in the cleavage of the O–O peroxo bond in PS (Figure 1a) and PMS (Figure 1b) to produce both sulfate and hydroxyl radicals. The sulfate radicals generation mechanism by photoelectrochemical activation for PS and PMS is represented in 8.

**Figure 1:**
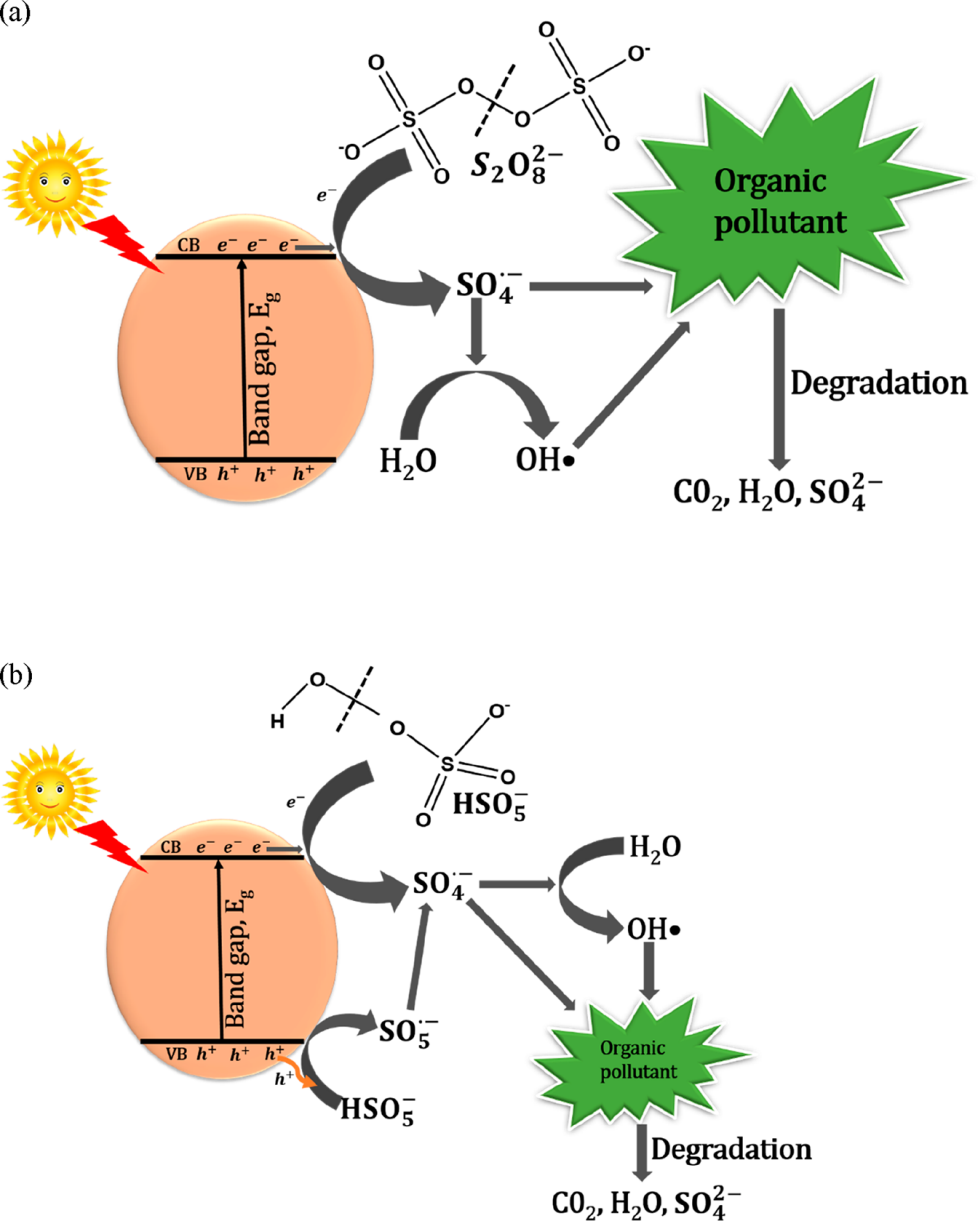
Possible mechanism for the activation of (a) PS and (b) PMS for degradation of organic pollutants.

Photoelectrochemical activation methods provide a platform for the generation of highly reactive oxidants such as photogenerated holes (eq 8), sulfate radicals (eq 9), and hydroxyl radicals (eq 10). It is worth noting that photoelectrochemical activation method is the only activation method that offers the possibility of producing photogenerated holes which are known to be a very strong oxidant and also produce sulfate radicals when compared to the conventional photoelectrochemical degradation technique which produces only photogenerated holes. Hence, harnessing the complementary oxidizing abilities of these highly reactive oxidizing species will result in improved degradation performance of the system. Thus, photoelectrochemical activation distinguishes itself as not just a promising activation method, but also a more efficient technique for wastewater treatment. Given that the photogenerated electron participates in the activation of persulfate ion to produce sulfate radicals (eq 9), the common challenge of recombination associated with semiconductors will be suppressed as the holes will have the mobility to react directly with the pollutants to degrade them (eq 11) and react with water molecules to generate hydroxyl radicals (eq 12).
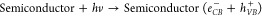
8
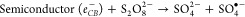
9
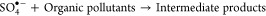
10
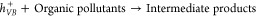
11
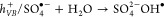
12
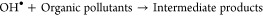
13

In addition, the sulfate radicals generation mechanism by photoelectrochemical activation for PMS is represented in 13.
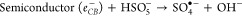
14
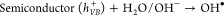
15
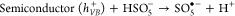
16
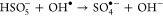
17
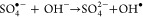
18
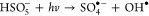
19
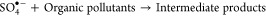
20
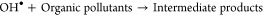
21

Several solar light driven semiconductor photocatalysts such as Cu_2_O,^52^ Bi_2_WO_6_,^53^ BiVO_4_,^54,55^ MoS_2_^56^ etc. have successfully been used to activate PS and PMS to produce sulfate radicals.

*In Situ Photoelectrochemical Activation.* The in situ generation of sulfate radical in photoelectrochemical degradation technology is a welcomed approach to avoid the burden and cost of adding more chemicals such as PMS and PS to the water treatment system. This approach can take advantage of the sulfate ion that is present in some industrial effluents^57^ and thus improve the potential of in situ photoelectrochemical activation for real life applications. Theoretically, when a semiconductor photocatalyst possessing valence band potential that is higher than the energy level of either sulfate ions or sulfate radicals is irradiated, photogenerated holes with the required oxidizing ability are produced. Therefore, there is electron transfer between the holes produced and sulfate ion to produce sulfate radicals. Minimal breakthrough has been recorded with this method as Li et al.^58^ reported the in situ photoelectrocatalytic production of sulfate radicals using BiPO_4_ modified with carbon paper in the presence of sodium sulfate as supporting electrolyte for improved degradation of pefloxacin. BiPO_4_ is a semiconductor photocatalyst with a higher valence band and conduction band potential above the energy level of most of the known free radicals including sulfate radicals. We envisage more reports on in situ generation of sulfate radicals.

The construction of a CoFe_2_O_4_/BiVO_4_ p–n heterojunction photoanode for photoelectrocatalytic peroxymonosulfate activation for environmental remediation was what Wang and co-workers^59^ devoted their attention to in their work. CoFe_2_O_4_ is a promising material with active sites for the photoelectrocatalytic activation of PMS owing to its improved catalytic ability arising from the coupling effect of cobalt and iron.^59^ To reduce the recombination tendency of CoFe_2_O_4_, Wang et al. prepared a heterojunction between CoFe_2_O_4_ and BiVO_4_ to allow the electrons the mobility to participate in PMS activation, and the holes to directly break down the organic pollutant. In the presence of pristine CoFe_2_O_4_ and PMS, the extent of tetracycline removal was found to be 49%, but in the absence of PMS the extent of tetracycline removal under photocatalytic, electrocatalytic and photoelectrocatalytic conditions were 12.5%, 8.2%, and 13.2% respectively (Figure 2). This result suggests two things: (i) CoFe_2_O_4_ is a suitable material for the photoelectrochemical activation of PMS, (ii) the presence of PMS enhanced degradation through the additional production of sulfate radical. Furthermore, their results show that the formation of heterojunction enhanced the degradation of tetracycline with percentage degradation of 64%, 68%, and 81% for BiVO_4_, CoFe_2_O_4_ and CoFe_2_O_4_/BiVO_4_ p-n heterojunction photoanode, respectively. Just as heterojunctions have improved the hydroxyl radical based PEC system,^60^ this work shows the semiconductor photocatalysts and the formation heterojunction can also be applied in sulfate radical generation.

**Figure 2:**
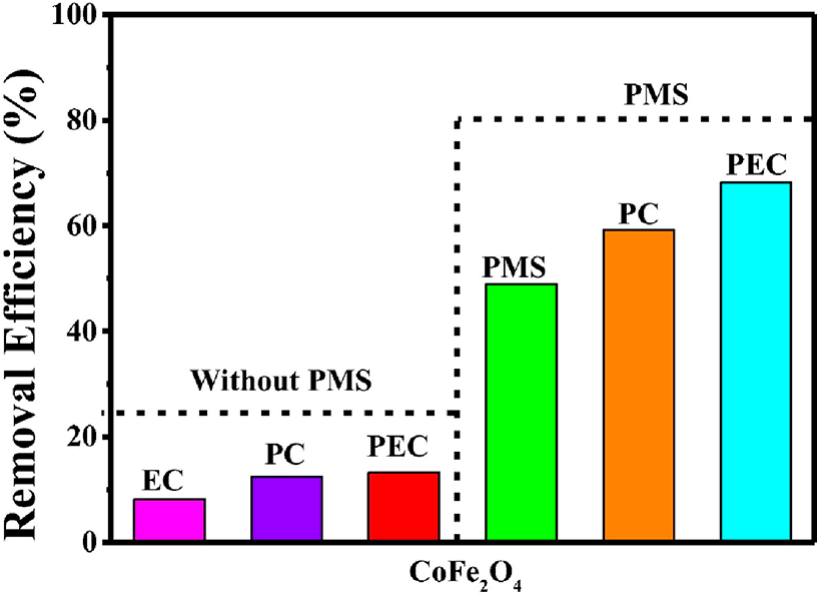
Degradation performances of photoanodes under different conditions: tetracycline concentration, 20 mg/L; bias potential, 0.6 V vs SCE, and [PMS] = 0.1 mM.^59^ Reproduced/adapted from ref (59). Copyright 2023 Elsevier.

Zheng et al.^61^ developed a highly efficient PEC/PMS system to break down norfloxacin with MoS_2_ doped with a carbon layer/carbon cloth as a PMS activator. They demonstrated the advantage of PEC/PMS activation over electrocatalytic and photocatalytic methods by the 100% norfloxacin degradation in 25 min against degradation of 43% and 52% from electrocatalysis and photocatalysis, respectively. This marked improvement in norfloxacin degradation by PEC/PMS can be attributed to the application of bias potential, facilitating the separation of the photogenerated electron and holes, thereby aiding the activation of PMS, leading to the generation of reactive oxidants for the removal of norfloxacin.^61^

The choice of a suitable photocatalyst that can be immobilized on a substrate to form a photoanode is the core of photoelectrochemical cells. It is a fact that the activity of semiconductor photocatalysts can be influenced by exposure to light, corrosion, stability, etc. To circumvent these drawbacks, other types of materials different from the conventional semiconductors such as materials based on metal organic frameworks (MOF), have been used to activate PMS. For example, Thamiselvan et al.^62^ prepared a zeolite imidazolate framework (ZIF) doped with bimetallic Ni/Co for the degradation of sulfamethoxazole. The results obtained in this report show the applicability of MOF in water treatment and PMS activation. This further opens the possibility that other types of materials may have the capability to cleave PMS for sulfate radical generation. The success recorded in this work further provides a solution to the shortcomings arising from PEC–PS/PMS activation using a semiconductor photocatalyst. In addition, this work also supports the recent trend of viewing wastewater as not just a waste but a resource for energy generation. Thamiselvan et al.^62^ showed this by designing a PEC/PMS system for dual purpose–pollutant degradation and green energy generation (Figure 3).

**Figure 3:**
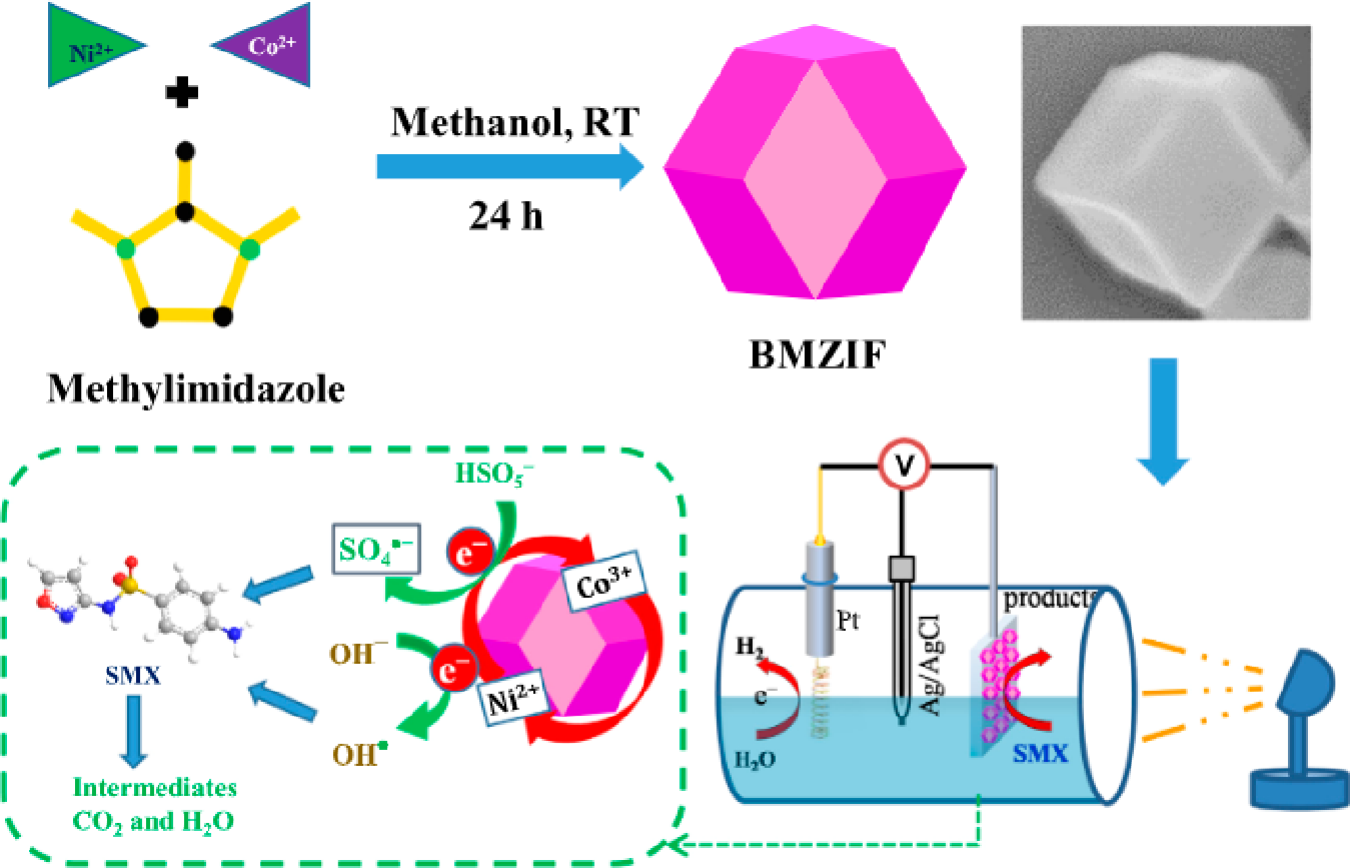
Preparation of BMZIF and photoelectrochemical degradation of SMX couple with H_2_ production.^62^ Reproduced/adapted from ref (62). Copyright 2023 Elsevier.

The homogeneous application of cobalt ion (in Co_3_O_4_ for example) for PMS activation in water treatment is hampered by the carcinogenicity or toxicity of the cobalt ion. To overcome this setback, Co_3_O_4_ can be immobilized on a photocatalyst. Decorating Co_3_O_4_ on a photocatalyst aids charge transfer via the Co^2+^ /Co^3+^ redox cycle, thus enhancing its catalytic performance and stability.^63^ For example, J. Li et al.^64^ immobilized Co_3_O_4_ on BiVO_4_ to form a heterojunction photoanode that was applied in the photoelectrochemical degradation of bisphenol – A (BPA).^64^ In the absence of PMS, pristine BiVO_4_ and Co_3_O_4_/BiVO_4_ gave rise to 13% and 21% BPA degradation, respectively. However, in the presence of 1 mM PMS, the extent of BPA degradation by Co_3_O_4_/BiVO_4_ photoanode anode rose to 96% which is about 4.5 times higher than in the absence of PMS. This marked increase, from their findings, was attributed to (i) successful immobilization of Co_3_O_4_ on BiVO_4_, (ii) improved charge separation and suppressed recombination rate facilitated by the p–n heterojunction formed, and (iii) the major contributions by key oxidants such as photogenerated holes, sulfate radicals and superoxide radicals. Thus, PEC activation of PMS is a mitigation of the challenge of cobalt poisoning in homogeneous activation.

The effect of PS in a PEC system using self-doped titanium dioxide nanotube arrays was investigated by Hong and co-workers.^65^ First, the extent of BPA degradation was interrogated under photolysis, electrocatalysis (EC), photocatalysis (PC), and photoelectrochemical oxidation (PEC) as shown in Figure 4.

**Figure 4:**
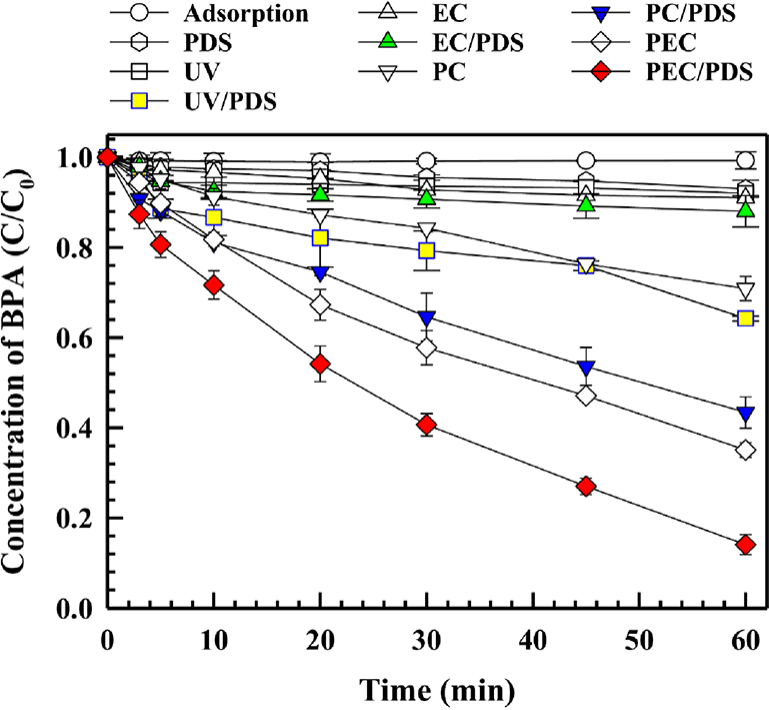
Removal efficiency of bisphenol A (BPA) using bl-TNAs under various operating conditions (photolysis, electrocatalysis [EC], photocatalysis [PC], and photoelectrocatalysis [PEC] with and without peroxydisulfate [PDS]; conditions [BPA]_0_ = 10.0 μM; [PDS]_0_ = 1.0 mM; [(NH_4_)_2_SO_4_]_0_ = 0.1 M; potential bias = 1.0 V vs Ag/AgCl; pH_*i*_ = 5.5).^65^ Reproduced/adapted from ref (65). Copyright 2021 Elsevier.

The result obtained confirms the possibility of PEC as a possible route for the activation of PDS due to the following: (i) under irradiation, there is a possibility of suppressed recombination of the photogenerated charge carriers by PS, leading to the generation of sulfate radicals, and (ii) since an increase in current density was observed in the presence of PS, it can be corroborated with the first reason that PS could bring about charge separation, thereby leading to BPA oxidation at the anode or production of hydroxyl radicals on the self-doped titanium dioxide nanotube arrays. Interestingly, electrochemical impedance spectroscopy showed a significant reduction in the *R*_ct_ value under PEC/PDS as compared to EC, EC/PDS, and PEC. This suggests that the PDS acts as an electron acceptor thereby enhancing the charge separation of the self-doped titanium dioxide nanotube arrays.

Second, the robustness and practical application of PEC/PDS were investigated by comparing results obtained using PEC in the absence and presence of PDS to treat pond water sample spiked with BPA, 4-chlorophenol, sulfamethoxazole, and carbamazepine. In the presence of 4 mM PS, a 5-fold increase in the extent of BPA degradation was reported as compared to the PEC system used in pond water only. In addition, the extent of PEC degradation increased in this order carbamazepine < sulfamethoxazole < 4-chlorphenol < BPA. Conclusively, the enhanced degradation in this system was due to the increased charge carrier separation and sulfate radicals.

Toward the improvement of the degradation of pollutants in photoelectrochemical systems, Orimolade et al. utilized a sulfate-assisted photoelectrochemical degradation at a Bi_2_WO_6_ anode.^66^ In the presence of Na_2_SO_4_, which is the widely used supporting electrolyte, the extent of sulfamethoxazole removal was 57% within 90 min. However, in the presence of 3 mM PMS, the degradation of sulfamethoxazole increased markedly to 98% within 90 min. The results obtained from the two systems showed that in the presence of PMS, the synergistic effect of the sulfate radicals and the photogenerated holes as well as the hydroxyl radicals significantly increased the extent of mineralization of the pollutant. To corroborate the above, scavenger studies showed that the photogenerated holes, sulfate radicals, and hydroxyl radicals contributed to the degradation process. Furthermore, the robustness of the sulfate-radical-based advanced oxidation process was studied toward other pharmaceutical pollutants. It was reported that the extent of degradation for tetracycline and diclofenac were 77% and 83% respectively. In addition, the specific energy consumption per unit TOC mass was evaluated and it was reported that the energy consumed in the photoelectrochemical PMS system (0.924 kWh g^–1^ of TOC) was lower than the energy consumed by the OH radical photoelectrochemical system (1.201 kWh g^–1^ of TOC). Overall, the PMS/PEC system was found to consume less energy and time, exhibit better versatility in degrading myriads of pharmaceutical pollutants, enhanced efficiency, etc. when compared to the OH radical photoelectrochemical system.

Several studies have shown that the loading of metals on photocatalysts as photoanode promotes the performance, stability, activation of PMS, and extent of degradation.^67−69^ The degradation of BPA increased with an increase in the concentration of PMS from 0 to 2 mM at PMS at a transition metal (cobalt) loaded BiVO_4_ photoanode.^54^ Furthermore, the effect of other transition metals such as nickel and iron when loaded on BiVO_4_ for PMS activation was studied. The PEC performance was found to follow this order: Co–BiVO_4_ > Fe–BiVO_4_ > Ni–BiVO_4_ > pristine-BiVO_4_. The outstanding performance displayed by the cobalt loaded system can be attributed to the standard reduction potential of Co^3+^/Co^2+^ which is 1.92 V as compared to Fe^3+^/Fe^2+^ which is 0.77 V and Ni^2+^/Ni which is −0.25 V. In addition, Co has a greater ability to recycle itself during catalysis reaction until all the PMS has been used up. Overall findings show the PEC/PMS Co–BiVO_4_ system to be an efficient wastewater treatment method for the remediation of organic pollutants.

Koiki et al. reported a novel approach to generating sulfate radicals from persulfate ion in a PEC system consisting of an FTO–Cu_2_O photoanode for the degradation of sulfamethoxazole.^52^ In the presence of 20 mM Na_2_SO_4_, the extent of sulfamethoxazole degradation was found to be about 36% within 2 h. However, in the presence of 10 mM Na_2_S_2_O_8_ and 20 mM Na_2_SO_4_, the extent of degradation increased to 72%. This significant increase is due to the presence of sulfate radicals resulting from the cleavage of persulfate ions. Interestingly, in the presence of 10 mM Na_2_S_2_O_8_ alone, the extent of degradation was found to be 84%. The difference reveals the possibility of some form of competition or sulfate radical quenching, thus resulting in insufficient sulfate radicals for degradation. Generally, bias potential is of great essence to drive the PEC system. Results from this work also showed that there was an increase in the percentage degradation of sulfamethoxazole on increasing the bias potential for the following reasons: (i) bias potential drives the electron toward the cathode, thus giving the photogenerated holes the mobility to react directly with the pollutant and degrade them. (ii) The photogenerated electrons can also react directly with the persulfate ion to generate sulfate radicals which are significantly responsible for driving the system. Furthermore, in this study, the total organic carbon content of the real wastewater sample containing sulfamethoxazole was reduced by nearly half of its initial value after 2 h. Thus, there is a significant contribution to the existing knowledge that visible light-driven semiconductors can also facilitate the generation of sulfate radicals for improved degradation of pollutants in a PEC system.

For real life applications, the addition of PS/PMS reagent may be mundane or add to the cost of treatment. Though we have critically discussed the ex situ generation of sulfate radicals and the prospect it brings to water treatment technology, we cannot rule away the fact that the introduction of PS/PMS into the system externally will not only increase the cost of treatment but poses a risk of giving rise to secondary pollution. This places a major concern on the ex situ generation of sulfate radicals. Interestingly, since sulfate ions are mostly present in industrial effluents and the sulfate concentration mostly found in surface water and industrial effluents ranges from hundreds of mg/L to thousands of mg/L respectively, it suggests the possibility of in situ generation of sulfate radicals from sulfate ions already present in the wastewater. This approach will cater for the setback of ex situ production of sulfate radicals and offer a cheaper and highly efficient wastewater treatment technology. Thus, the generation of sulfate radical in situ, just like OH radical will be a welcome approach. In this light, Yuan and co-workers demonstrated the possibility of activating sulfate without the use of external chemical oxidants.^58^ Sulfate radicals are generated in situ (from the constituent of the wastewater) at a BiPO_4_ semiconductor. BiPO_4_ has sufficient redox capacity to generate hydroxyl radicals, superoxide radicals, and sulfate radicals. Therefore, upon irradiation under UV light, sulfate radicals are produced according to eqs 22–27 .
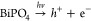
22
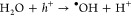
23
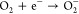
24
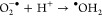
25
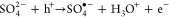
26
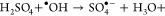
27

Their findings showed that the extent of PEC degradation of perfloxacin was 5.1 times higher than PC, 8.2 times higher than electrochemical degradation, and 3.3 times higher than that in the absence of sulfate radicals. This marked enhancement in PEC is due to the synergistic effects of light, bias potential, photocatalysis, and electrochemical degradation. Electron spin resonance spectra and scavenger studies strongly corroborated the fact that sulfate radicals, hydroxyl radicals, and superoxide radicals were generated in the SR-PEC system, and they played an active role in the degradation process.

Table 2 highlights other reports on PS/PMS assisted PEC for wastewater treatment.

**Table 2:** Studies on Photoelectrochemical Degradation of Organic Pollutants in the Presence of PS and PMS

Materials	Salt	Analyte	% removal	Salt concentration	Ref
CoFe_2_O_4_/BiVO_4_	PMS	Tetracycline	89.1% within 60 min	0.1 mM PMS	(59)
1T/2H-MoS_2_@C/CC	PMS	Norfloxacin	100% within 25 min	40 mg/L PMS	(61)
BMZIF	PMS	Sulfamethoxazole	100% after 24 min	0.1 mM PMS	(62)
Co_3_O_4_/BiVO_4_	PMS	Bisphenol-A	96% within 120 min	1 mM PMS	(64)
BiVO_4_–PDA/CF	PMS	Ofloxacin	100% within 120 min	2 mM PMS	(70)
bI-TNAs	PS	Bisphenol-A	85.9% within 60 min	1 mM PS	(65)
Bi_2_WO_6_	PMS	Sulfamethoxazole	98%	3 mM PMS	(66)
		Diclofenac	83%		
		Tetracycline	79% within 90 min		
Co–BiVO_4_	PMS	Bisphenol-A	99.16% within 60 min	2 mM PMS	(54)
Cu_2_O	PS	Sulfamethoxazole	86% within 120 min	10 mM PS	(52)
BiPO_4_/CP	N/A	Pefloxacin	87.8% in 120 min	N/A	(58)
BiVO_4_	PMS	Bisphenol-A	100% within 120 min	5 mM PMS	(55)
γ-Bi_2_MoO_6_/Cu	PS	Diclofenac	86.3% within 120 min	10 mM PS	(71)
Co_3_O_4_/CFP	PMS	Phenol	100% within 120 min	2 mM PMS	(72)
bI-TNA	PS	Bisphenol-A	93.4% within 60 min	4 mM PS	(73)
MnFe_2_O_4_/CFP	PMS	Bisphenol-A	∼100% within 90 min	1 mM PMS	(74)
TiO_2_/WO_3_	PMS	17α-ethinyl estradiol	88.8% within 60 min	10 mg/L PMS	(75)
NCD@CNFO	PMS	trimethoprim	97% within 60 min	0.1 mM PMS	(76)

This article discusses the electrochemical, photoelectrochemical, and in situ photoelectrochemical generation of sulfate monoanion radicals for the oxidation/degradation of organic pollutants in water or wastewater. The reported works have shown the potential contribution of sulfate radical generation to improving PEC performances. Based on these reports, we put forward the following perspectives:

(i) The possibility of the photoelectrochemical approach to sulfate radicals generation, especially via solar light driven materials, opens up more room for further research in the design of photoanodes and systems in SR-PEC for water treatment. We expect to see more investigations and insight on the production of sulfate radicals by different photoanodes fabricated from a myriad of visible light active semiconductors for greener water treatment methods. We believe that the various methods of improving the photoelectrochemical performance of semiconductor(s) in hydroxyl radical generation can also be applied to sulfate radical generation while noting the peculiarity in the bandgap levels of the materials.
(ii) The higher oxidative power and longer lifetime of sulfate radical in comparison to hydroxyl radical can be harnessed to improve the performance of photoelectrochemical processes.
(iii) The role of sulfate dianions and PS/PMS in the reduction of electron–hole recombination rate is still not well understood and thus can be explored further.
(iv) More research should be focused on in situ generations of sulfate radicals instead of the general method of production of radicals that involve PS or PMS salts, which could result in a large amount of sulfate ions in our water systems and an increase in cost. The in situ generation will be useful in treating sulfate rich wastewater.
(v) Since we do not have reports on electro- or photoelectroactivation of persulfate for the treatment of wastewater treatment such as landfill leachate and industrial wastewater. Attention should be given to this area by researchers as we explore the possibility of lending the application of sulfate radical advanced oxidation process to real life applications.
(vi) The few available reports on SR-PEC seem to favor the fact that the overall degradation performance of the system is improved by the presence of sulfate radicals. For sulfate radical generation to be more widely accepted, more understanding is needed of its performance in more complex wastewater matrices. The associative, competitive, and inhibitive performances of sulfate radical when in combination with other radicals need to be studied in depth.

The low volume of work on SR-PEC and SR-AOP in general suggests that this area of research is still in its infancy. Thus, we believe that more attention should be focused on this area to advance research on sustainable alternative/complementary wastewater treatment methods.
